# Feasibility, acceptability, and preliminary effects of PATH FOR timely transfer of geriatric HIP fracture patients from hospital to rehabilitation to home (PATH4HIP): a protocol for a mixed method study

**DOI:** 10.1186/s40814-022-01079-z

**Published:** 2022-06-11

**Authors:** Chantal Backman, Anne Harley, Steve Papp, Colleen Webber, Stéphane Poitras, Randa Berdusco, Paul E. Beaulé, Veronique French-Merkley

**Affiliations:** 1 School of Nursing, Faculty of Health Sciences, University of Ottawa; Affiliate Investigator, Ottawa Hospital Research Institute, Affiliate Investigator, Bruyère Research Institute, 451, Smyth Road, RGN 3239, Ottawa, ON K1H 8M5 Canada; 2 Bruyere Continuing Care; Assistant Professor Faculty of Medicine, University of Ottawa, 43 Bruyère St, Ottawa, ON K1N 5C8 Canada; 3 Faculty of Medicine, University of Ottawa, The Ottawa Hospital, General Campus, 501 Smyth Rd, Ottawa, ON K1H 8L6 Canada; 4 Ottawa Hospital Research Institute, 501 Smyth Rd, Ottawa, ON K1H 8L6 Canada; 5 School of Rehabilitation Sciences, Faculty of Health Sciences, University of Ottawa, 451, Smyth Road, Ottawa, ON K1H 8M5 Canada; 6 Division of Orthopaedic Surgery at The Ottawa Hospital, Faculty of Medicine, University of Ottawa, General Campus, 501 Smyth Rd, Ottawa, ON K1H 8L6 Canada; 7 Faculty of Medicine, University of Ottawa, 43 Bruyère St, Ottawa, Ontario K1N 5C8 Canada

**Keywords:** Hip fractures, Geriatric rehabilitation, Older adults, Transitions in care

## Abstract

**Background:**

Hip fractures in older adults are significant contributors to severe functional decline and disability as well as hospitalization and increased health care costs. Research shows that timely referral to geriatric rehabilitation leads to better patient outcomes. Currently, a wide variability in the timing, the frequency, and the choice of appropriate setting for rehabilitation of hip fracture patients exists.

**Aim:**

Evaluate the feasibility, acceptability, and preliminary effectiveness of PATH4HIP, a pathway intervention for timely transfer of post-operative geriatric hip fracture patients from hospital to rehabilitation to home.

**Methods:**

This is a single-arm, pragmatic feasibility study to measure reach, effectiveness, adoption, implementation, and maintenance of PATH4HIP, a pathway for post-operative hip fracture patients from a large academic health science center to a geriatric rehabilitation service in Ottawa, Canada. During a 6-month period, all hip fracture patients, 65 years of age or older who have undergone surgery and have met the eligibility criteria (*n* = 96), will be transferred to the geriatric rehabilitation service no later than post-operative day 6. Patients (*n* = 10–12) and clinicians who are working on the orthopedic team (*n* = 10–12) and on the geriatric rehabilitation service (*n* = 10–12) will be invited to participate in an interview to share their feedback on the intervention’s feasibility and acceptability and to provide suggestions to improve PATH4HIP. Descriptive statistics will be used to summarize results of the quantitative data and content analysis will be used to analyze the qualitative data. The study will be open for recruitment from January to July 2022.

**Discussion:**

If feasible, PATH4HIP will result in the reduction of the post-operative acute care length of stay to less than or equal to 6 days, while having no detrimental effect on rehabilitation outcomes such as functional gains, or discharge destination.

**Supplementary Information:**

The online version contains supplementary material available at 10.1186/s40814-022-01079-z.

## Background

Hip fractures in older adults are significant contributors to severe functional decline and disability as well as hospitalization and increased health care costs [1–4]. In Canada, approximately 30,000 hip fractures occur annually with one hip fracture currently costing on average $37,500 in 1-year costs across multiple sectors [5]. These health care costs have been reported as comparable to those of other countries [6–8].

Best practice guidelines recommend standardized processes, including early post-operative mobilization [1, 9–11]. Specifically, hospital care pathways should adopt the goal of active rehabilitation starting no later than 6 days after the patient’s surgery [1]. Inpatient geriatric rehabilitation is considered the gold standard and represents a critical component of post-operative hip fracture care when striving to maximize functional recovery [12]. Geriatric rehabilitation involves a multidisciplinary set of evaluative, diagnostic, and therapeutic interventions with the aim of restoring functional ability and enhancing residual functional capability in elderly individuals with disabling impairments [13, 14]. One systematic review of 17 trials (*n* = 4780) demonstrated that inpatient rehabilitation specifically targeted at geriatric patients improved outcomes related to function, improved outcomes related to admission to nursing homes, and decreased mortality rates [15]. Furthermore, another study found that geriatric hip fracture programs were associated with savings from the perspective of health and social services and were more effective than usual care in reducing length of stay, improving function, and increasing the rate of returning home after discharge [16]. Studies have also demonstrated shortened acute care length of stay without compromising rehabilitation outcomes for hip fracture patients [17–22].

Despite this, many hospitals are still facing challenges in meeting these targets. Evidence suggests that delays to the start of rehabilitation exist as well as a wide variability in timing, frequency, and choice of appropriate setting for rehabilitation [2]. The purpose of our study is to pilot PATH4HIP, a pathway intervention for timely transfer of post-operative geriatric hip fracture patients from hospital to rehabilitation to home. Our specific objectives include to (1) determine the feasibility and acceptability of the PATH4HIP intervention; (2) evaluate PATH4HIP’s preliminary effects on acute and rehabilitation length of stay, functional gains in rehabilitation, and discharge destination; and (3) refine methods in preparation for a larger trial.

## Methods

**Fig. 1:**
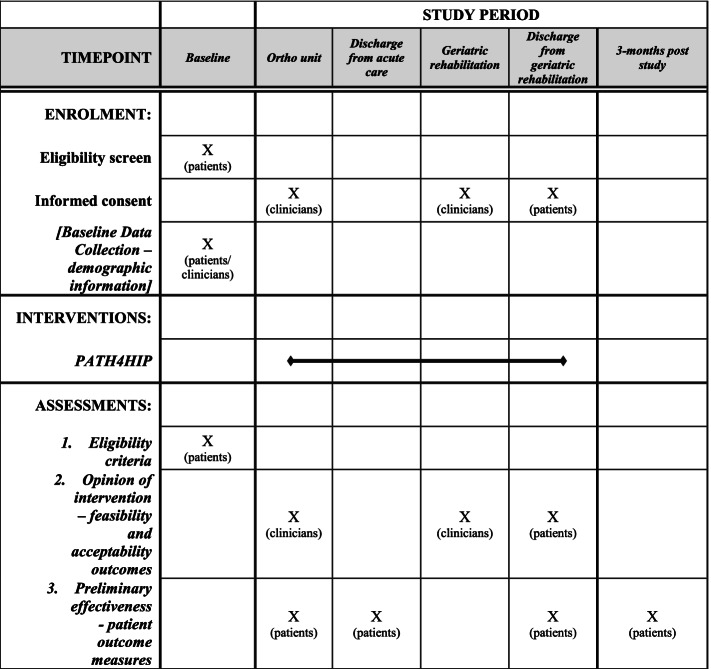
Standard Protocol Items: Recommendations for Interventional Trials (SPIRIT) figure of enrolment, interventions, and assessments

This study is approved by the Ottawa Health Science Network Research Ethics Board (#20180469-01H), the Bruyère Research Ethics Board (#M16-18-036), and the University of Ottawa Research Ethics Board (#H-08-18-1061). The protocol is formulated in accordance with the SPIRIT (Standard Protocol Items: Recommendations for Interventional Trials) statement (see Additional file 1) [23] (Fig. 1). This protocol is version 3.0, dated of January 4, 2022. The study is scheduled to open for recruitment in January 2022.

### Study design and setting

This is a single-arm, pragmatic feasibility study using the RE-AIM framework [24] to measure REACH (how to reach the intended population), EFFECTIVENESS (how to tell if the intervention is effective), ADOPTION (how to develop organizational support to deliver the intervention), IMPLEMENTATION (how to ensure the intervention is delivered properly), and MAINTENANCE (how to incorporate the delivery long-term).

The study will take place in a large academic health science center and on a geriatric rehabilitation service of a complex continuing care organization in Ottawa, Ontario, Canada.

### Intervention

**Fig. 2:**
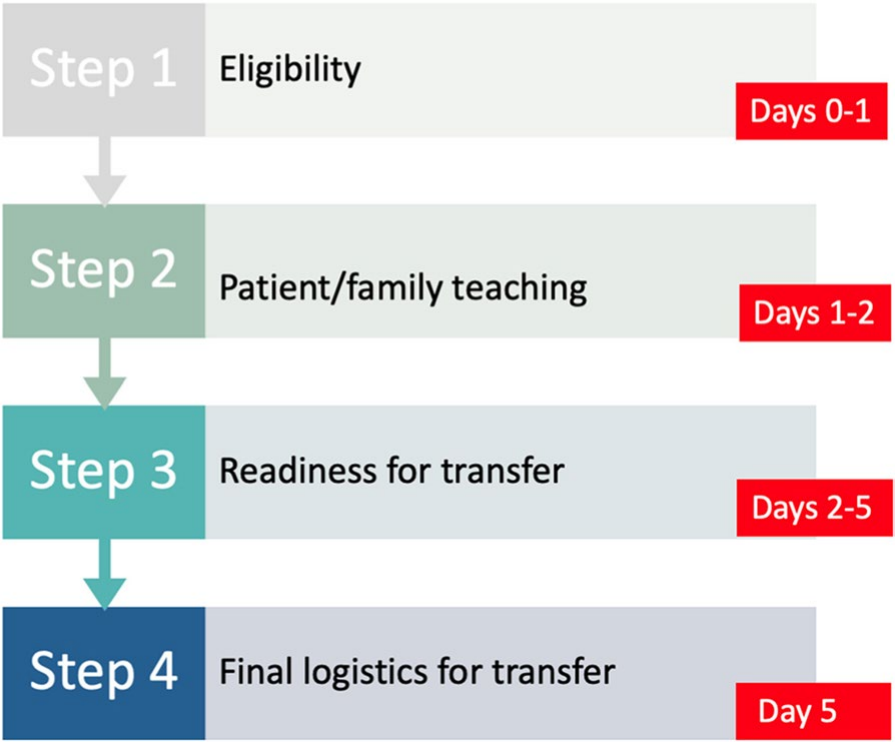
Basic visual representation of the PATH4HIP steps

The intervention is described using the Template for Intervention Description and Replication (TIDieR) guidelines [25]. PATH4HIP, the pathway for timely referral of geriatric hip fracture patients to rehabilitation, adheres to the best practice guidelines that mandate that all hip fractures require rehabilitation [1, 9–11]. All patients who meet the defined eligibility criteria will be accepted and transferred to geriatric rehabilitation no later than post-operative day 6. PATH4HIP is an approach to help identify, prioritize, and refer geriatric hip fracture patients and consists of four steps: *Step 1:* Identify eligible patients and obtain consent; *Step 2:* Provide patient and family teaching; *Step 3:* Determine readiness for transfer; and *Step 4:* Finalize logistics for transfer. Figure 2 is a basic visual representation of the PATH4HIP steps.

The roles of each team member during the post-operative days 1–5 are defined as follows:

#### Step 1—Identify eligible patients and obtain consent

##### Post-operative days 0–1

A senior nurse or delegate will review the daily census through the electronic chart data report and will identify patients that meet the preliminary inclusion criteria. The hospitalist will also support in identifying eligible patients. The orthopedic resident will determine weight bearing status and range of motion restrictions post-surgery. The senior nurse will confirm patient consent for rehabilitation and will initiate the referral to the subacute team. A geriatric rehabilitation physician will be available to answer questions regarding eligibility and medical stability.

##### Post-operative days 1–3

Nurses will communicate the patient status for eligibility at daily discharge rounds. Physiotherapy will collaborate with the team to establish baseline ambulation and to facilitate the patient consent process. The subacute consult assessor will verify that the patient meets the inclusion criteria. The clinical admission coordinator at the geriatric rehabilitation service will liaise with the subacute team to obtain the status of eligible patients.

#### Step 2—Provide patient and family teaching

##### Post-operative days 1–3

Clinical nurses will provide the PATH4HIP patient and family education materials (e.g., pamphlet and video) and will document in the patient’s chart once completed. The subacute consult assessor will meet with the eligible patient and will explain the geriatric rehabilitation program in more detail.

##### Post-operative days 3–5

The clinical team will reinforce rehabilitation best practices and answer questions from patients and families. The orthopedic resident will support the team’s recommendations for the patient and for the family regarding appropriate rehabilitation needs.

#### Step 3—Determine readiness for transfer

##### Post-operative days 2–5

The senior nurse will communicate with members of the team at discharge rounds regarding readiness for transfer. Clinical nurses will update the patient status (delirium, wound, pain, etc.) at discharge rounds. The physiotherapist will provide an update on the patient’s mobility status at discharge rounds. The hospitalist will evaluate each patient daily for delirium and medical stability to promptly address any issues. The hospitalist will also provide feedback to the team regarding the medical stability of the patient. The orthopedic resident will monitor patients for surgical post-op complications.

#### Step 4—Finalize logistics for transfer

##### Post-operative days 3–5

The subacute consult assessor will complete the rehabilitation referral and will flag priority for the geriatric rehabilitation admission team. The senior nurse will communicate with the clinical admission coordinator in the geriatric rehabilitation program. The senior nurse will also communicate with the clerk to book the transport to geriatric rehabilitation. The manager of patient flow in geriatric rehabilitation will make themselves available should any challenges need to be addressed.

The implementation strategy, informed by behavioral change techniques [26] from our previous barriers and enablers’ analysis [27], consists of flags in the clinical pathway*,* standardized transfer of information, high-risk delirium screening, dashboard to provide immediate feedback to clinicians, patient information, multidisciplinary workshops, and reminders and sustained site engagement. Further information about the implementation strategy is described in Table 1.

**Table 1:** Implementation strategy

Behavioral change techniques [26]	Mode of delivery (how)	Content (what materials, what procedure)	Provider (who provided)	Setting (where)	Recipient (to whom)	Intensity (when and how much) over how many contacts)	Duration (over what period of time)
**Prompts/cues** (introduce stimulus with the purpose of prompting or cueing the behavior)	**Flags in the clinical pathway**	Key reminders will be prompted (i.e., anticipated discharge date on or before day 6)	Manager and/or the physician assistant	Ortho and off service units	Nurses, occupational therapists, physiotherapist, social workers, physicians, residents, administrators	Ongoing	Ongoing
	**Standardized transfer of information (**to include all essential patient information)	Key information needed for referral will be sent to geriatric rehabilitation	Admission coordinator	Ortho and off service units	Nurses, occupational therapists, physiotherapist, social workers, physicians, residents, administrators	Ongoing	Ongoing
	**High-risk delirium screening**	Identify high risk patients for delirium	Manager and/or the physician assistant	Ortho and off service units	Nurses, occupational therapists, physiotherapists, social workers, physicians, residents, administrators	Ongoing	Ongoing
**Feedback on behavior** (monitor and provide feedback on the outcome of performance of the behavior)	**Dashboard to provide immediate feedback to clinicians**	Provide data to all team members (for example: # of hip fractures, % referred to geriatric rehabilitation, etc.)	Research assistant	Ortho, off service units and geriatric rehabilitation	Nurses, occupational therapists, physiotherapist, social workers, physicians, residents, administrators	Weekly	Ongoing
**Social and environmental consequences** (provide information (e.g., written, verbal, visual) about social and environmental consequences of performing the behavior)	**Multidisciplinary workshops** ^a^	PATH4HIP training Provide evidence to staff about the advantages of discharging patients earlier	Educators	Ortho, off service units and geriatric rehabilitation	Nurses, occupational therapists, physiotherapist, social workers, physicians, residents, administrators	Workshops	60 min
	**Reminders** ^a^ (i.e., posters, daily rounds)	Provide visual, verbal and written reminders to all team members	Local champions	Ortho, off service units and geriatric rehabilitation	Nurses, occupational therapists, physiotherapist, social workers, physicians, residents, administrators	Ongoing	5–10 min
	**Sustained participant and site engagement** ^a^ (including site visits, emails to all staff and physicians)	Discuss progress, and improvement opportunities	Local champions	Ortho, off service units and geriatric rehabilitation	Nurses, occupational therapists, physiotherapist, social workers, physicians, residents, administrators	Regular follow-ups with all participants at all sites	15–30 min
	**Patient information** 1) Paper booklet 2) Information on organizations’ website 3) Video	Educate patients about benefits of going to geriatric rehabilitation, what to expect, etc.	Educators	Ortho, off service units and geriatric rehabilitation	Patients and families	Ongoing	5–10 min

^a^In order to maintain fidelity of our intervention delivery, all multidisciplinary workshops and presentations will be facilitated by one or more of the researchers, all of whom will undergo training to promote consistency in delivery methods and content (how well planned)

### Sample size

The primary outcome is to estimate feasibility, which will be determined by study adherence. In this proposed 6-month study, we expect a total sample of 96 participants (16 eligible individuals per month × 6 months). With a sample size of 96 participants, proportion of 75% of eligible participants who are transferred to geriatric rehabilitation by post-operative day 6 could be estimated to within a 95% confidence interval of ± 8.7% [28–31].

### Eligibility

The inclusion criteria are as follows: patients (1) aged 65 and older with a unilateral hip fracture, (2) anticipated discharge to community, (3) post-operative hemoglobin greater than 70 g per liter, (4) consult services signed off for acute medical issues, and (5) pre-fracture ambulating independently with or without gait aid. The exclusion criteria are as follows: (1) pathologic fracture, metastatic cancer diagnosis, (2) on dialysis, chemotherapy, and/or radiation treatment, (3) living in long-term care, and (4) acute agitated delirium.

### Data collection

The study of the use of PATH4HIP will have three stages: (1) introductory stage, (2) 6-month of PATH4HIP use, and (3) qualitative and quantitative evaluation.

1. Introductory stage

All clinicians who are working on the orthopedic unit and on the geriatric rehabilitation service will be invited to attend multidisciplinary workshops to introduce PATH4HIP. The research assistant will track all clinicians approached and clinician attendance. Following the workshops, participants will be asked to complete a survey including sociodemographic information (age, gender, professional role, clinical unit, and years in practice), their opinion on the intervention, and any feedback that could be used to improve PATH4HIP.

(2) Six-month of PATH4HIP use

A daily census list of all potential hip fracture patients will be generated. The unit manager (or a senior nurse) and a subacute care assessor on the orthopedic unit will apply the PATH4HIP eligibility criteria. Patients who are eligible will be enrolled in PATH4HIP. A trained research assistant will track all patients who are included, all patients who are excluded, including the reasons for exclusion, and all patients who refuse to go to geriatric rehabilitation along with their reasons for refusal. The research assistant will also track the adherence to all steps of PATH4HIP, the reasons for any non-adherence, and the completion status of PATH4HIP.

(3) Qualitative and quantitative evaluation

Prior to discharge from geriatric rehabilitation*,* the research assistant will obtain informed consent and conduct 60-min audio recorded semi-structured interviews with patients (*n* = 10–12) in person or by video call to gather their opinion and to gather specific information on what can be improved to enhance their experience. Clinicians who are working on the orthopedic team (*n* = 10–12) and on the geriatric rehabilitation service (*n* = 10–12) will be invited to participate in an audio-recorded 30–45-min interview to share their feedback on the intervention’s feasibility and acceptability and to provide suggestions to improve PATH4HIP.

Using administrative databases, we will collect the following information for all unilateral hip fracture patients during the study period to compare participants and non-participants: age, gender, ethnicity, relationship status, living situation, Elixhauser score, comorbidities, total acute length of stay, post-surgery length of stay, discharge destination, return to the emergency department (30 days), readmissions (90 days), geriatric rehabilitation length of stay, functional gains, and discharge destination post geriatric rehabilitation.

### Outcomes

Evaluation will be based on feasibility, acceptability, and preliminary effects following the RE-AIM framework [24] as follows:

*REACH*: proportion of individuals who participate, proportion of excluded patients, proportion of patient refusals, reason for excluded patients and refusals, proportion of clinicians approached to participate in the workshops, proportion of clinicians who attended the workshops, and clinicians’ opinion on the intervention following the workshops

*EFFECTIVENESS*: acute care and rehabilitation length of stays (median), % discharged back to community, % transfer back to acute care and reason, and proportion of functional gains in rehabilitation. The Functional Independence Measure (FIM) [32] will be used to calculate the difference between the patients’ functional status at rehabilitation admission and discharge. The FIM is a disability measure that consists of 18 items each rated on a scale of 1 (most dependent) to 7 (most independent) according to the degree of assistance required to perform a specific activity in six domains: self-care, sphincter control, mobility, locomotion, communication, and social cognition. The total FIM score ranges from 18 to 126. The FIM consists of two major criteria, the motor FIM (score 13–91), and cognitive FIM (score 5–35) [32]

*ADOPTION*: proportion of patients that completed PATH4HIP

*IMPLEMENTATION*: proportion of hip fracture geriatric patients referred from acute care to geriatric rehabilitation, proportion of patients who are discharged by post-op day 6, proportion of patients where intervention was delivered as per the study protocol, reasons for any deviations, adaptations made to intervention during study, and average cost of intervention (time and money)

*MAINTENANCE*: patients’ opinions on the intervention: ease of use of the patient information, relevance of information, follow-up with clinicians including their opinion of the content and its relevance, and follow-up with administrators on organizational plans for PATH4HIP

Dimensions of REACH, ADOPTION, IMPLEMENTATION, and MAINTENANCE will be assessed to answer objective 1 (feasibility and acceptability of the PATH4HIP intervention) and dimension of EFFECTIVENESS will be assessed to answer objective 2 (PATH4HIP’s preliminary effects). All data sources, methods, and outcome/process measures for each dimensions of the RE-AIM framework [24] are further described in Table 2.

**Table 2:** RE-AIM evaluation of PATH4HIP [24]

Dimensions	Questions addressed	Data sources	Methods	Outcome or process measures
**REACH (who)**	To what extend did PATH4HIP reach the intended population?	Patients Research staff	Study logs and unit census	Feasibility and acceptability outcomes **Proportion of individuals who participate** (proportion of eligible patients approached to participate, proportion of eligible patients who accepted to participate in PATH4HIP) **Exclusion** (proportion of excluded patients, proportion of patient refusals) **Barriers to recruitment** Reason for excluded patients, and refusals
		Workshops for clinicians	Workshop invitation emails	Feasibility and acceptability outcomes **Proportion of clinicians approached to participate in the workshops** **Proportion of clinicians who attended the workshops**
			Survey following the workshops	Feasibility and acceptability outcomes **Clinicians’ opinions on the intervention**
**EFFECTIVENESS (what)**	How effective was the intervention?	Patients	Administrative databases	Preliminary effects **Outcomes (quality indicators)** (acute care and geriatric rehabilitation length of stays (median), proportion of functional gains in rehabilitation (admission and discharge functional independence measure (FIM) [32]), % discharged back to community, % transfer back to acute care and reason)
**ADOPTION (where)**	To what extent was the intervention adopted by target individuals?	Clinicians Research staff	Study logs	Feasibility and acceptability outcomes **Proportion of patients that completed PATH4HIP**
**IMPLEMENTATION (how)**	How consistent was the implementation? What adaptions were made along the way?	Research staff Clinical manager and physician assistant	Research records, informal interviews with clinical manager and physician assistant	Feasibility and acceptability outcomes **Proportion of hip fracture geriatric patients referred from acute care to geriatric rehabilitation** **Proportion of patients who are discharged by post-op day 6** **Adherence to PATH4HIP** (proportion of patients where intervention was delivered as per the study protocol (all steps completed)) Reasons for any deviations **Adaptations made to intervention during study** **Average cost of intervention—time** **Average cost of intervention—money**
**MAINTENANCE (when)**	Are there plans to include the pathway as part of the organizational programs?	Patients	Semi-structured interviews	Feasibility and acceptability outcomes **Patient experience with PATH4HIP (opinion of the intervention)** Patient information: ease of use, relevance of information, experience
		Clinicians	Interviews	Feasibility and acceptability outcomes **Follow-up with clinicians on content and relevance of PATH4HIP** Overall opinion on the intervention Opinion of the value of the intervention (benefit) Perceived burden of the intervention Feasibility to perform the intervention Barriers to performing or implementing the intervention Suggestions for improvement of the intervention
		Administrators	Meetings	Feasibility and acceptability outcomes **Follow-up with leadership on organizational plans for PATH4HIP**

### Data analysis

#### Quantitative analysis

##### Sociodemographic information

We will use appropriate descriptive statistics (mean and standard deviation for normally distributed continuous variables [e.g., age], median and interquartile range for skewed continuous variables [e.g., acute care and rehabilitation length of stays], frequency and proportion for categorical variables [e.g., type of rehabilitation service]). We will also describe and compare the participants and non-participants’ sociodemographic and clinical information during the study period.

##### Objective 1 (feasibility and acceptability of the PATH4HIP intervention)

All outcomes under the dimensions of REACH, ADOPTION, and IMPLEMENTATION will be measured using proportions and reported with a 95% confidence interval.

##### Objective 2 (PATH4HIP’s preliminary effects)

We will use descriptive statistics for outcomes under the dimension of EFFECTIVENESS as follows. For normally distributed continuous data, we will use mean and standard deviation (e.g., % discharged back to community, % transfer back to acute care). For skewed continuous data, we will report median and interquartile range (e.g., acute care and rehabilitation length of stays). For categorical data, we will report frequency and proportion [e.g., functional gains in rehabilitation (admission and discharge functional independence measure (FIM) [32]). All data analysis will be performed with IBM SPSS Statistics version 23.

#### Qualitative analysis

*Objective 1 (feasibility and acceptability of the PATH4HIP intervention)*: All interviews will be transcribed verbatim. Two researchers will independently code the transcripts, and we will perform coding and content analysis on the transcripts using an iterative process until consensus on the coding and content analysis is reached [33]. The qualitative data analysis software (NVivo, QSR International) will be used to manage all the qualitative data.

#### Data management

The principal investigator will oversee all aspects of the study. All data will be stored in accordance with the research ethics board procedures and requirements for storage and security of data. Hardcopy files will be kept securely in a locked filing cabinet. Electronic files will be stored on a secure server and files with participant identifying information will be password protected. Participants will be allocated a unique study participant identification number, which will be used in any documentation associated with the study. Audio recordings of the interviews will be destroyed after verbatim transcripts have been prepared. Only the study investigators and research assistants will have access to the study data. Ethics auditing procedures will be determined by the research ethics board. Research records will be stored for a period of 10 years.

#### Progression criteria

Progression to a larger follow-up study will be determined by the following criteria:

1. At least 75% of patients that are deemed eligible for PATH4HIP agree to participate
2. At least 75% of the participants are transferred to geriatric rehabilitation by post-operative day 6

Based on the CONSORT guidelines for pilot and feasibility studies [28], we will report against these criteria using a traffic light system: red (not to proceed), amber (proceed with amendments), or green (proceed).

## Discussion

Study results will inform future work to test the effectiveness of the intervention across multiple sites. If successful, this approach has the potential to be spread across multiple settings for hip fracture patient transitions from acute care to rehabilitation.

### Strengths and limitations

This study has several strengths. The intervention was developed from a systematic barriers and enablers analysis [27] using the Theoretical Domains Framework [34, 35]. Currently, most quality improvement approaches tend to lack a theoretical base for conceptualizing clinical decision-making and behavior change processes. This often makes it difficult to apply emerging evidence and spread change across a variety of health care settings. In contrast, our approach designed based on the results of a theory-based barriers and enablers analysis [27] allowed us to study the challenges of hip fracture transfer from acute care to rehabilitation from the perspectives of clinicians, administrators, senior leaders, patients, and families. PATH4HIP was then designed using a prioritization analysis of behavior change techniques [26] that we will implement in this current study in order to support the adherence and sustainability of the intervention. Our theory-informed intervention will also allow us to select monitoring metrics that can be replicated in other organizations and will assist in discerning the mechanisms behind how and why an intervention succeeds or fails. Our design of PATH4HIP and its implementation strategy have emphasized cross-sectoral collaboration, between acute and subacute care. Most importantly, this study also prioritizes best practices for hip fracture outcomes and embeds them at the heart of the intervention design, using a low-cost solution.

This proposed study is a single-arm, pragmatic feasibility study and therefore will also have some limitations due to the lack of a control group. Although timely access to rehabilitation is a best practice for geriatric hip fracture patients [1, 9–11], it is possible that we will need to adapt the intervention during the study period due to unanticipated consequences. We will document these changes as they may inform the methods required for a larger study to evaluate PATH4HIP across multiple sites.

## Supplementary Information

**Additional file 1.** The Standard Protocol Items Recommendations for Trials (SPIRIT) checklist.

## Data Availability

No additional data are available.
